# An iron-base oxygen-evolution electrode for high-temperature electrolyzers

**DOI:** 10.1038/s41467-023-35904-7

**Published:** 2023-01-17

**Authors:** Kaifa Du, Enlai Gao, Chunbo Zhang, Yongsong Ma, Peilin Wang, Rui Yu, Wenmiao Li, Kaiyuan Zheng, Xinhua Cheng, Diyong Tang, Bowen Deng, Huayi Yin, Dihua Wang

**Affiliations:** 1 School of Resource and Environmental Sciences, Wuhan University, Wuhan, 430072 China; 2 Hubei International Scientific and Technological Cooperation Base of Sustainable Resources and Energy, Wuhan, 430072 China; 3 School of Civil Engineering, Wuhan University, Wuhan, 430072 China

**Keywords:** Corrosion, Materials for energy and catalysis, Chemical engineering, Electrocatalysis

## Abstract

High-temperature molten-salt electrolyzers play a central role in metals, materials and chemicals production for their merit of favorable kinetics. However, a low-cost, long-lasting, and efficient high-temperature oxygen evolution reaction (HT-OER) electrode remains a big challenge. Here we report an iron-base electrode with an in situ formed lithium ferrite scale that provides enhanced stability and catalytic activity in both high-temperature molten carbonate and chloride salts. The finding is stemmed from a discovery of the ionic potential-stability relationship and a basicity modulation principle of oxide films in molten salt. Using the iron-base electrode, we build a kiloampere-scale molten carbonate electrolyzer to efficiently convert CO_2_ to carbon and oxygen. More broadly, the design principles lay the foundations for exploring cheap, Earth-abundant, and long-lasting HT-OER electrodes for electrochemical devices with molten carbonate and chloride electrolytes.

## Abstract (web summary)

A low-cost and efficient high-temperature oxygen evolution reaction electrode is a big challenge. Here, the authors report an iron-base electrode with an in situ formed lithium ferrite for enhanced stability and catalytic activity in molten carbonate and chloride salts and achieve kiloampere-scale electrolysis.

## Introduction

Electrifying materials and chemicals by electrolysis is becoming a golden key to reduce greenhouse gas emissions owing to the ever-increasing renewable power-generated electricity^1–7^. Among various electrolysis devices, high-temperature molten-salt electrolyzers are able to make significant contributions to our modern society due to their favorable reaction kinetics. The Hall-Héroult process for aluminum production and other molten salt electrolyzers for reactive metals (magnesium, lithium and etc.) are well-established examples. In addition, molten chloride electrolysis^1,2,8,9^ and molten carbonate electrolysis^10–12^ have been intensively studied in recent years with the aim to convert metal oxides and CO_2_ to value-added metal/alloys and carbon/carbon-based fuels at high temperature. However, a low-cost and long-lasting HT-OER electrode is still absent because of the serious materials degradation under anodic polarization^13,14^.

A satisfactory HT-OER electrode should have good electrical conductivity, catalytic performance, corrosion-resistance, and mechanical robustness^13^. Although precious metals/alloys or their oxides are commonly used as OER electrode materials in laboratory^6,15^, they are impractical for large-scale HT-electrolyzers due to the scarcity. Alternatively, low-cost transition metals (TMs) with a conductive and protective oxide scale could be a promising HT-OER electrode material^16–20^. Fe, Ni, Cu, and their alloys have been widely investigated as the anode material, and many important achievements have been made^21,22^. For instance, the nickel-base alloys exhibited good stability and oxygen evolution performance in high-temperature molten carbonates^23,24^, but were corroded seriously in low-temperature molten carbonates^25^ and molten chlorides^21,26^. The addition of La, Ti, Al could improve the stability of nickel-base alloy anode^25,26^. But the long-term stable HT-OER electrode was not achieved yet due to the lack of understanding on the stability of anodically polarized oxides in high-temperature molten salts. In addition, the anodic oxidation behaviors of typical metals have not been systematically studied along with the basic physicochemical properties of these metals and their oxides.

To mitigate the big challenge, here we establish an ionic potential-stability relationship of a series of oxides that can set a guideline for selecting stable oxides in various molten salts. In addition, we find a new method to solve the serious corrosion problem caused by Cl^−^ via increasing the basicity of the in situ formed protective oxide layer. Based on the discoveries, we develop an iron-base electrode with an in situ formed LiFe_5_O_8_ layer (lithium ferrite oxide, LFO) that is able to effectively catalyze oxygen evolution reaction in ternary molten carbonate and molten LiCl–Li_2_O electrolyzers. Moreover, theoretical calculations show that the LFO film has good mechanical compatibility, strong interface adhesion, and high diffusion energy barriers for Cl^−^.

## Results and discussion

### Ionic potential-oxide stability relationship

**Fig. 1:**
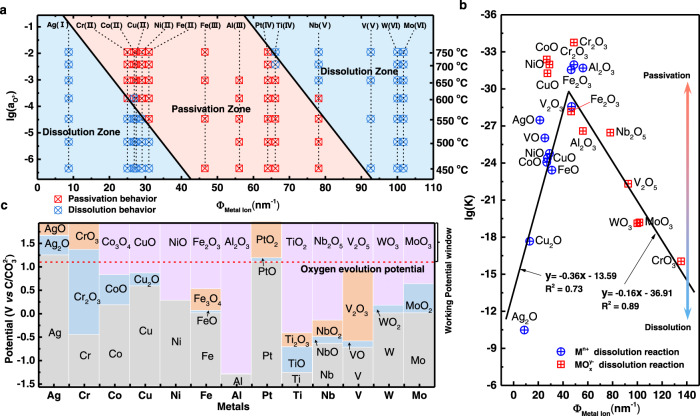
Criteria for the selection of oxides for HT-OER electrodes. **a** Anodic oxidation behaviors of typical metals (Ag, Cr, Co, Cu, Ni, Fe, Al, Pt, Ti, Nb, V, W, and Mo) as a function of their corresponding ionic potentials (*Ф*_Metal Ion_) in molten Li_2_CO_3_–Na_2_CO_3_–K_2_CO_3_ with different *lg(*${a}_{{{{{{\rm{O}}}}}}^{2-}}$*)*, the region can be divided into dissolution and passivation zones, circle, and square shapes respectively represent the dissolution and passivation_._ **b** Equilibrium constants of typical metal oxide dissolution reactions as a function of their corresponding ionic potentials (650 °C). **c** Potentials of the formation of metal oxides and stabilization potential window of oxides.

We first measured the anodic polarization curves of a series of pure metals (Ag, Cr, Co, Cu, Ni, Fe, Al, Pt, Ti, Nb, V, W, and Mo) to reveal the correlation between anodic behavior and basicity of molten Li_2_CO_3_–Na_2_CO_3_–K_2_CO_3_ at 450–750 °C (*B*_ms_: −6.34 to −1.95) (Supplementary Figs. 3–5). The basicity of molten salt is defined by Eq. (1) and it increases with the operating temperature (Supplementary Notes 1, Supplementary Fig. 3, and Supplementary Table 1).

1 $${B}_{{{{{{\rm{ms}}}}}}}=\,{lg}({{a}}_{{{{{{\rm{O}}}}}}^{2-}})$$

where *B*_ms_ stands for the basicity of molten salt and ${a}_{{{{{{\rm{O}}}}}}^{2-}}$ is the activity of O^2−^. The anodic stability of metals is closely related to the *B*_ms_ and the properties of metals (Supplementary Table 3). In general, the anodic dissolution of metal electrode depends on the solubility of the anodic oxidation products. Learning from the classical theory of ionic potential of different metal ions in aqueous solutions, the solubility of a metal compound in a specific solution correlates with the ionic potential of the metal ion^27^. For example, iron oxides can dissolve in hydrochloric acid while titanium dioxide cannot because the ionic potential of titanium (IV) ion is stronger than that of iron ions. Cation ionic potential (*Ф*) is an indicator of the charge density at the surface of a cation, which is the ratio of the charge number (*n*) with the ion radius (*R*), reflecting the cation polarization power^28^. The *Ф* of typical metal cations is listed in the Supplementary Table 4. Combining the ionic potentials and the anodic dissolution behaviors of typical metals studied in this work, we find that the dissolution of oxides in molten carbonate is related to both ionic potential and *B*_ms_, which can be divided into three regions: two dissolution regions and one passivation region (Fig. 1a). The oxides of *Ф* = 0–40 tend to be more stable in a more basic electrolyte, the oxides of *Ф* = 40–65 are stable in a wide *B*_ms_ range, and the oxides of *Ф* = 65–110 tend to be more stable in a more acidic electrolyte. The differences of the two dissolution regions represent two kinds of dissolution products: M^n+^ and MO_*x*_^*y*−^. As shown in Fig. 1b, the equilibrium constants of the metal oxide dissolution reactions agree well with the dissolution behaviors in Fig. 1a, i.e., a smaller equilibrium constant means that the corresponding oxide is more stable. Besides a low solubility, the oxide should withstand a strong anodic polarization to allow the fast oxygen evolution on the electrode. As shown in Fig. 1c, the oxide that is stable above the OER potential line meets the standard of enabling the OER. For example, Fe_2_O_3_, NiO, CuO, Co_3_O_4_, TiO_2_, Nb_2_O_5_, V_2_O_5_, WO_3_, and MoO_3_ are stable, while Cr_2_O_3_ and PtO are not stable at the potential higher than OER potential. Consequently, using this principle, we can choose a certain metal that forms an insoluble and thermodynamically stable metal oxide in a given molten salt, and the solubility of the oxide scale can be mediated by controlling the *B*_ms_.

### Iron-base HT-OER electrode in molten carbonates

**Fig. 2:**
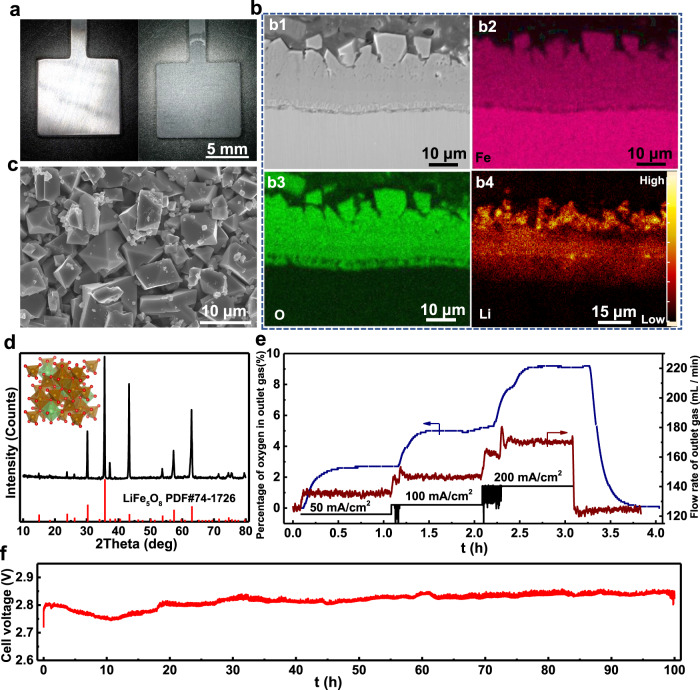
HT-OER performances of the Fe electrode in molten carbonates. **a** Optical graphs of the iron anode before and after pre-oxidation in molten carbonates. **b** SEM image (b1), EDS mapping (Fe (b2), O (b3)), and SIMS mapping (Li (b4)) of the cross-section of oxide scale. **c** SEM image of the oxide scale that directly contacts with molten salt. **d** XRD pattern of the oxide scale. **e** Profiles of the oxygen content of the outlet gas as functions of current density and time during constant-current electrolysis. **f** Cell voltage profile as a function of time at 100 mA/cm^2^ in molten Li_2_CO_3_–Na_2_CO_3_–K_2_CO_3_ at 450 °C.

Based on the above-established ionic potential-solubility relationship, we selected iron as the target HT-OER electrode. In our previous work, the Ni10Cu11Fe alloy had been proven as a stable HT-OER electrode in molten carbonate^24^. However, this electrode cannot survive in molten carbonate when the operating temperature is below 500 °C (*B*_ms_ < −5.34)^10^. According to Fig. 1a, Ni(II) and Fe(II) oxides stay in the dissolution region. The reason that causes dissolution of protective oxides scale can be explained to low *B*_ms_ of molten carbonate at these temperatures. Fortunately, Fe(III), Pt(IV), Al(III), Ti(IV), and Nb (V) oxides are in the passivation region when the temperature is lower than 500 °C (Fig. 1a). However, Al(III), Ti(IV) and Nb(V) oxides are insulators that cannot serve as the OER electrode. In terms of considering the cost, only Fe(III) oxide stays in the passivation region in a wide range of *B*_ms_. To avoid the generation of Fe(II) during the electrochemical oxidation of Fe, we pre-oxidized the iron electrode in molten Li_2_CO_3_–Na_2_CO_3_–K_2_CO_3_ at 650 °C at which temperature Fe(II) oxide is stable (Fig. 1a) and the Fe(II) can be subsequently oxidized to Fe(III) under selected anodic potentials (Fig. 1c). Thus, an Fe(III) oxide film forms after pre-oxidation, and the oxide film has a regular octahedrons structure (Fig. 2a, c). To our surprise, the oxide film is dense and electronically conductive, which is radically different from the Fe(III) oxide that is commonly generated from the high-temperature oxidation^29^. According to XRD and EDS analysis, the oxide film consists of LiFe_5_O_8_, which was generated by the reaction of Fe_2_O_3_ and Li_2_CO_3_ (Fig. 2b, d). LiFe_5_O_8_ is a good electrical conductor^30^. The achieved LFO film has a surface electrical resistance of 0.12 Ω cm^2^ at 450 °C (Supplementary Fig. 6a), and is thermodynamically stable and insoluble in molten salt in a wide temperature range (450–750 °C, Supplementary Fig. 5i). Thus, the artificially prepared Fe(III) oxide solves the dissolution problem caused by the in situ formed Fe(II) oxide that is soluble in molten Li_2_CO_3_–Na_2_CO_3_–K_2_CO_3_ under 550 °C. This result strongly supports the proposed ionic potential-oxide stability relationship. In addition, the density functional theory (DFT) calculation results show that the extracted bulk (shear) moduli of Hill average for Fe and LiFe_5_O_8_ are 196.0 (83.1) and 206.2 (58.6) GPa and the calculated adhesion energy of the interface is 3.39 J/m^2^_,_ indicating the good mechanical compatibility and strong interface adhesion of Fe and LiFe_5_O_8_ (Supplementary Notes 2). Hence, the incorporation of Li_2_O into the Fe(III) oxide further improves both electrical conductivity, density, and mechanical robustness of the protective oxide film.

Oxygen gas generated from the pre-oxidized Fe electrodewas detected at different current densities. During electrolysis, a lot of oxygen bubbles evolved from the electrode were observed from a quartz window during the high temperature electrolysis (Supplementary Video). The oxygen generating rate are 9.8 mmol/h at 50 mA/cm^2^, 19.6 mmol/h at 100 mA/cm^2^, 41.1 mmol/h at 200 mA/cm^2^, and the corresponding faradic efficiencies are 95.3, 95.3, and 99.9% (Fig. 2e). The anodic polarization curve shows that the iron anode coated with a LFO film has no obvious oxidation current before reaching the oxygen evolution potential of 1.1 V and possesses an equivalent current density in comparison with the commercial Ti/IrO_2_–Ta_2_O_5_ anode (IR-MMO, Magneto Special Anodes Co. Ltd.) over the potential range of 1.1–1.9 V (Supplementary Fig. 6b). Moreover, the iron electrode works stably for 100 h at 100 mA/cm^2^ (Fig. 2f) and the cell voltage profile stays around ~ 2.8 V with no obvious fluctuations, indicating its good electrochemical durability and catalytic stability. After electrolysis, the dimension of the anode and the composition of the oxide film maintains its original shape and chemical composition (Supplementary Fig. 6c–g). We further measured the iron concentration in molten salt electrolyte and cathodic product. The iron concentrations in the obtained carbon product and electrolyte are 680 and 15 ppm, respectively. Based on the iron concentration, the consumptions rate of the Fe electrode is 0.07 cm per year, which is much slower than the standard value of the inert anode in the aluminum electrolyzer^18^.

In addition, a kiloampere-scale electrolyzer (Li_2_CO_3_–Na_2_CO_3_–K_2_CO_3_, 650 °C) with an oxygen-evolution anode (iron with the LFO film) was built to convert CO_2_ to carbon and O_2_ in our lab (Supplementary Fig. 7a). Using this kiloampere-scale device, we can produce 2.7 kg of carbon materials and 5.0 Nm^3^ of O_2_ per day (Supplementary Fig. 7b). Note that the product selectivity is 99.0% along with a faradic efficiency of 87.6% and energy consumption of 35.0 kWh/kg-carbon. Although the system energy consumption could be higher than this value, this is a big step towards bringing the proof-of-concept research from the laboratory to commercial products. Among various CO_2_ reduction electrolyzers, scaling up the electrolyzer is still challenging. As such, we design and run this kiloampere-scale electrolyzer in hope of pushing this technology close to the market and making a real societal impact.

### Iron-base HT-OER electrode in molten LiCl–Li_2_O

**Fig. 3:**
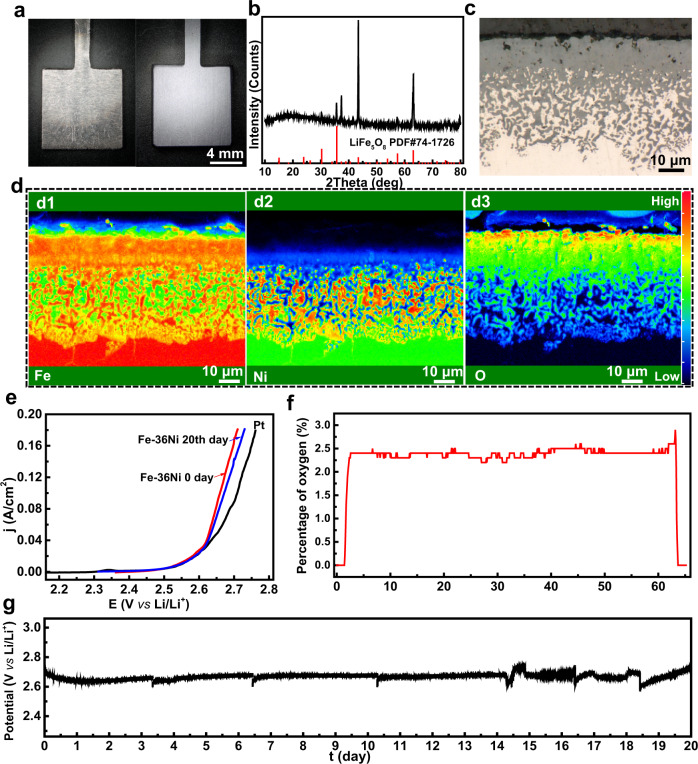
HT-OER performances of the Fe–36Ni electrode in molten LiCl–Li_2_O at 650 °C. **a** Optical graphs of the Fe–36Ni anode before (left) and after pre-oxidation(right). **b** XRD pattern of the oxide scale. **c** Optical micrograph of the cross-section of oxide scale. **d** EPMA mapping of the cross-section (d1: Fe, d2: Ni, d3: O) of the oxide scale. **e** Anodic polarization curves in molten lithium chloride (5 mV/s). **f** Profiles of oxygen content of the outlet gas as functions of time during constant-current electrolysis. **g** Potential profile as a function of time at 100 mA cm^−2^ in molten LiCl–1.5wt.%Li_2_O at 650 °C.

We further tested the stability of the lithium ferrite oxide layer for OER in molten LiCl–Li_2_O, which has been intensively studied for recycling MO_*x*_ spent nuclear fuels^31^. The long-existing challenge of developing low-cost metal OER electrodes in chloride ion-containing electrolytes is the super corrosivity of the molten chloride^32^. Since the operating temperature of LiCl–Li_2_O is higher than 600 °C, the Fe–36Ni alloy was chosen because Ni can increase the resistance of high-temperature oxidation (Supplementary Figs. 8–11)^33^. Like the pure iron electrode, a dense oxide film is formed on the Fe-36Ni electrode after pre-oxidation (Fig. 3a, c). The 15 μm-thickness oxide film consists of LiFe_5_O_8_, whose component is the same as that formed at the iron electrode (Figs. 3b and 3d). Note that Ni oxide is not found in the oxide film because the diffusion rate of Fe in the bulk alloy is faster than Ni. Because of the different diffusion rates of Fe and Ni, a nickel-rich transition layer forms between the oxide layer and the bulk metal, which increases the high-temperature oxidation resistance and mechanic stability of the protective layer (Supplementary Fig. 12).

The HT-OER performance of the pre-oxidized Fe–36Ni electrode was evaluated in molten LiCl–Li_2_O. As shown in Fig. 3e, the OER of the Fe–36Ni and platinum electrode start at the same potential and have similar Tafel slopes, indicating that the Fe-36Ni electrode has a favorable OER kinetics. After 20 days’ service, the polarization curve maintains the same shape as the original Fe–36Ni electrode, suggesting its high stability (Fig. 3e). In the meantime, we observed oxygen gas evolution from the electrode, further confirming that OER happened at the Fe–36Ni electrode (Fig. 3f). More importantly, the Fe–36Ni electrode continuously works as the OER electrode for 20 days with a stable potential of 2.7 V (Fig. 3g). After electrolysis, the composition of Fe–36Ni electrode does not change and the thickness of the oxide film increases only by 7 μm (Supplementary Fig. 13), further demonstrating its super stability. According to the iron concentration in molten salt (19 ppm) and cathodic product (15.8 ppm), the calculated consumption rate is about 0.04 cm per year, which is the lowest consumption rate of low-cost metallic inert anode in molten halogen melt so far^9,22^.

### Protection mechanism in chloride melt

**Fig. 4:**
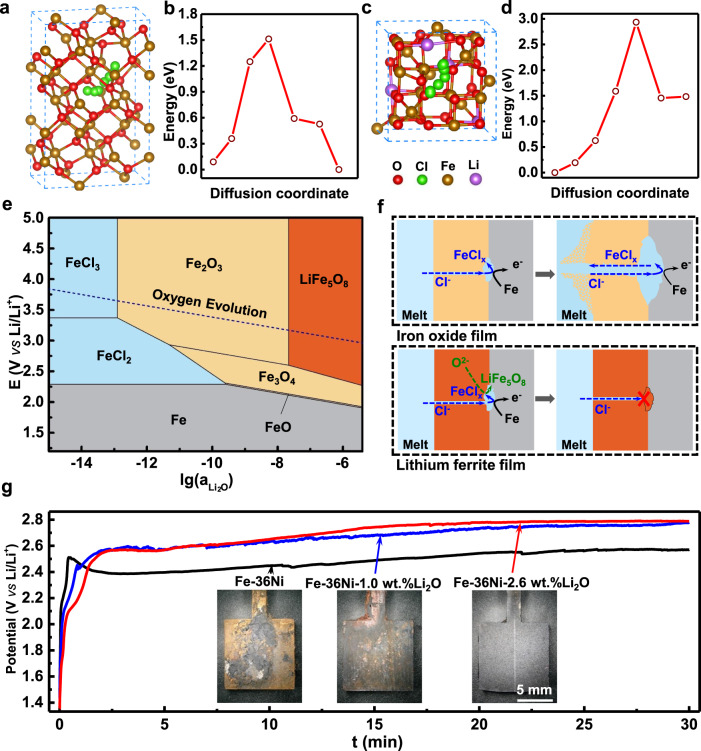
Protection mechanism of the LFO film in molten chloride. **a** Cl migration paths in Fe_2_O_3_. **b** Cl migration energy profile in Fe_2_O_3_. **c** Cl migration paths in LiFe_5_O_8_. **d** Cl migration energy profile in LiFe_5_O_8_. **e** E-lg$({{{\mbox{a}}}}_{{{{\mbox{Li}}}}_{2}{{\mbox{O}}}}){{\mbox{}}}$ diagram of iron (650 °C, Supplementary Notes 3). **f** The proposed self-repair mechanism of the lithium ferrite film. **g** Potential profiles of Fe-36Ni, Fe-36Ni-1.0wt.% Li_2_O, Fe-36Ni-2.6wt.% Li_2_O electrodes at 100 mA/cm^2^ in molten LiCl-1.5wt.% Li_2_O, the inset pictures are the electrodes after electrolysis.

The surprisingly stable LFO film in molten LiCl–Li_2_O drives us to rethink the protection mechanism of the film in the highly corrosive environment. As we know, the Cl^−^-induced corrosion is difficult to preclude because Cl^−^ can easily combine with metal ions to form soluble chlorides. Thus, preventing the contact of Cl^−^ with metal could be a solution to solve the active corrosion problem. In this regard, the stability of the iron-base electrode may be attributed to the LFO film that can effectively stop Cl^−^ entering and reaching the metal substrate. The density functional theory (DFT) calculation results of Cl^−^ diffusion through Fe_2_O_3_ and LiFe_5_O_8_ are given in Fig. 4a–d. The migration energy barrier for Cl^−^ in LiFe_5_O_8_ (2.93 eV) is much higher than that in Fe_2_O_3_ (1.51 eV), which might be understood by the higher atomic density of LiFe_5_O_8_ (0.11 atom/Å^3^) than that of Fe_2_O_3_ (0.09 atom/Å^3^)(Supplementary Notes 2). The high migration energy of Cl^−^ in the LiFe_5_O_8_ indicates the lower diffusion rate of Cl^−^ in LFO film, suggesting that LiFe_5_O_8_ is a good barrier to prevent Cl^−^ penetration. In addition to the barrier effect, the chemical environment is also helpful to prevent the pitting corrosion caused by Cl^-^. As shown in Fig. 4e, LiFe_5_O_8_ prefers to generate when increasing the concentration of Li_2_O. Since molten LiCl has a high concentration of Li_2_O, LiFe_5_O_8_ is the thermodynamically stable species if the Li_2_O concentration is high. Moreover, LiFe_5_O_8_ can liberate O^2−^ more easily than Fe_2_O_3_. This means that the LFO film has an alkaline environment, where Cl^−^ cannot bond with iron ions to form soluble FeCl_2_ or FeCl_3_ (Fig. 4e, f). Hence, the basicity of the oxide film repels Cl^-^ and thereby avoid the combination of iron ions with Cl^−^. Even some Cl^-^ can attack the substrate, the alkaline environment will convert iron chlorides to thermodynamically more stable iron oxide.

We further verified this basicity-regulated self-repair process by adding Li_2_O into the alloy to tune the basicity of the oxide film. As shown in Fig. 4g, the bare alloy electrode forms porous oxide film and the potential of the electrode does not reach the OER potential (2.6 V vs Li/Li^+^), indicating that the anodic reaction is the oxidation of metals rather than the OER. The electrode become more stable when adding 1.0wt.% Li_2_O in the alloy, confirming that increasing the basicity of oxide film increases the stability of the electrode. However, this oxide film still has some corrosion pits. As expected, the formed oxide film is dense when the alloy contained 2.6wt.% Li_2_O. Thus, increasing the basicity of the oxide film indeed improves the stability of the oxide electrode. In short, the basicity-self repair process is governed by the basicity of the oxide film that prevents the attack from Cl^−^ and endows the self-repair capability, thereby achieving the long-term stability in high-temperature molten halides.

We invented a durable iron-base HT-OER electrode with an in situ formed LiFe_5_O_8_ scale that can serve in both molten carbonate and LiCl-Li_2_O. The revealed ionic potential-stability relationship of a library of oxides lays a foundation for screening suitable oxides that are chemically stable and insoluble in molten salts with different basicities. To solve the ultra-high corrosivity of Cl^−^, we control the basicity of LiFe_5_O_8_ to induce the liberation of O^2−^ that can repel the Cl^−^ and thereby prevent the attack from Cl^−^. DFT calculations further reveal that the LiFe_5_O_8_ is an effective barrier to prevent Cl^−^ attack with a diffusion energy barrier of 2.93 eV. The rationally designed iron-base electrode with enhanced basicity can work stably in molten LiCl–Li_2_O for 20 days, which outperforms the state-of-the-art low-cost HT-OER electrodes. Therefore, the results reported herein sets a paradigm for exploring low-cost and long-lasting HT-OER electrodes for molten-salt electrolyzers, especially for molten carbonates and chlorides, aiming to expedite the electrification of green materials synthesis and thereafter close the carbon cycle. In addition to enabling zero-emission electrolyzers, the design principles and underlying mechanism set a paradigm for exploring durable corrosion-resistant materials under extreme conditions, and the oxygen product is an essential feedstock for outer space exploration using local resources.

## Methods

### Alloy electrode preparation

The metal powders of nickel (99.9 wt.%, 500 mesh) and iron (99.9 wt.%, 500 mesh), 1 mm-thickness iron plates (99.9 wt.%) and Fe–36Ni alloy were purchased from Sinopharm Chemical Regent Co., Ltd. The Fe electrode and Fe–36Ni electrode were prepared by cutting iron plates and Fe–36Ni alloys into plates with a size of 10 mm × 10 mm × 1 mm. The Fe–36Ni–Li_2_O electrodes were fabricated by Fe and Ni powders with addition of different concentration of Li_2_O powders (1.0 and 2.6 wt%), which were mixed proportionally by ball milling for 3 h and then was made into rods using spark plasma sintering (SPS) at 35 MPa and 960 °C. The as-prepared rods were cut into plates (10 mm × 10 mm × 3 mm) by wire-electrode cutting.

### Electrochemical tests in molten carbonates

An alumina crucible (8 cm OD, Shanghai Shuocun) containing 1000 g of anhydrous Li_2_CO_3_–Na_2_CO_3_–K_2_CO_3_ (mixed in a molar ratio of 43.5: 31.5: 25.0) was used as the molten salt bath. The mixed salt was first dried at 200 °C for 24 h and melted at 800 °C. The crucible was protected by argon-1vol.% carbon dioxide mixture gas flow. After that, the temperature was adjusted to different working temperatures. The reference electrode was a graphite rod (3 mm in diameter) inserted in a closed one-end mullite tube filled with 2 g of Li_2_CO_3_–Na_2_CO_3_–K_2_CO_3_ (molar ratio of 43.5: 31.5: 25.0). The counter electrode was a 50 cm^2^ nickel sheet, and the working electrode was metal wires electrodes (1 mm in diameter). Linear sweep voltammetry was conducted at a sweep rate of 5 mV/s on CHI1140 electrochemical workstation (Shanghai Chenhua Instrument Co. Ltd., China). Pre-oxidation was performed by constant-current electrolysis between the iron anode (10 mm × 10 mm × 1 mm/30 mm × 30 mm × 1 mm) and the nickel cathode at 100 mA/cm^2^ at 650 °C. Electrolysis was performed between pre-oxidized iron anodes and nickel cathodes at 100 mA/cm^2^ under CO_2_ atmosphere at 450 °C. The current was supplied by a battery testing machine (Neware Technology Limited, China).

### Anodic gas analysis

A U-shape titanium tube (6.5 cm OD) was filled with 1800 g of molten carbonates. The anode and cathode were respectively immersed into the two sides of the U-shape electrolyzer (Supplementary Fig. 14). This use of U-shape electrolyzer can effectively avoid the gas crossover between the anode and cathode. The flow rate of argon gas was 125 mL/min at both sides of the U-shape electrolyzer. The depth of the pre-oxidized iron anode (30 mm × 30 mm × 1 mm) in the molten salt was 3.5 cm. The electrolysis was conducted at 50 mA/cm^2^, 100 mA/cm^2^, 200 mA/cm^2^, respectively. The outlet gas from the anode side was detected by a gas analyzer (AGA1000, Aut. EQ. co. Ltd, China).

### Observing the OER

The OER was directly observed using a transparent cell containing a 10 cm × 10 cm quartz window. A pyrex glass crucible (7.5 cm × 7.5 cm × 20 cm) was used to contain 500 g of pre-melting Li_2_CO_3_–Na_2_CO_3_–K_2_CO_3_ (mixed in a molar ratio of 43.5: 31.5: 25.0) (Supplementary Fig. 15). Note that this crucible only worked stably at a temperature below 500 °C. Electrolysis was conducted at 100 mA/cm^2^ between a pre-oxidized iron anode (20 mm × 20 mm × 3 mm) and a nickel plate cathode. Illumination was provided by a supplementary lamp from the rear window, and the video was taken from the front window by a camera.

### Electrochemical tests in molten LiCl–Li_2_O

A Ni crucible containing 500 g of anhydrous LiCl was first dried at 200 °C for 24 h and then melted at 650 °C in an argon atmosphere. Afterwards, Li_2_O was added into LiCl, and the concentration of Li_2_O was kept at 1.5 wt.%. Polarization curves were obtained using a three-electrode system containing a pre-oxidized Fe–36Ni alloy working electrode, an Ni/NiO reference electrode, and a NiO cathode contained at the bottom of the Ni crucible. The pre-oxidized Fe–36Ni alloy electrode was prepared at 750 °C in a two-electrode system containing the above-mentioned molten carbonate, the Fe36Ni anode, and the nickel cathode (100 mA/cm^2^ for 11 h). The electrolysis was conducted at 100 mA/cm^2^ for 20 days at 650 °C. To control the basicity of the oxide film, different amounts of Li_2_O were added into Fe-36Ni alloys prepared by the SPS sintering process. The concentrations of Li_2_O were controlled at 0, 1.0, and 2.6 wt.%. Note that the Fe–36Ni–Li_2_O electrodes were directly employed as the anode without pre-oxidation.

### Characterizations

Before characterizing, all samples were washed in deionized water to remove the adhered electrolyte and dried in a vacuum furnace at 80 °C for 12 h. Phases of oxide film were identified by X-ray diffraction (XRD, Shimadzu X-ray 6000 with Cu Kα1 radiation at λ = 1.5405 Å). The optical photos of both surfaces and cross-sections of all electrodes before and after electrolysis were recorded by a high-magnification optical microscope ((Keyence VHX-5000). The SEM (TESCAN, Mira3 LMH), SEM-EDS (Oxford, Aztec Energy X-Max 20), EPMA (JEOL JXA-8530FPlus), time-of-flight secondary ion mass spectrometry (TOF-SIMS, PHI TRIFT-II) were used to investigate element distributions of the cross-sectional surface.

### DFT calculation

The calculations were performed by using first principles calculations with Vienna Ab-initio Simulation Package (VASP)^34,35^. The Perdew-Burke-Ernzerhof (PBE) was employed to describe the electron exchange and correlation^36^. While the plane wave cutoff energy 500 eV was used in all calculations to ensure accuracy. Monkhorst–Pack scheme was adopted for the k-point sampling of Brillouin zone integration with the density > 30 Å^37^. The lattice parameters and atom positions of all calculations were relaxed until the convergence of the force below 0.01 eV/Å. For calculation of the adhesion energy of Fe and LiFe_5_O_8_, the cell of Fe was compressed according to the cell of LiFe_5_O_8_ with misfit 2.3%. Then, Fe and LiFe_5_O_8_ were optimized with a vacuum over 18 Å for preventing the artificial interaction between the periodic replicas. Finally, the optimized LiFe_5_O_8_ (001) and Fe (100) were put together with the nearest atoms over 0.2 nm before optimization. All atoms were able to relax during optimization, the distance between LiFe_5_O_8_ (001) and Fe (100) is less than 0.2 nm after reaching convergence of the force below 0.01 eV/Å. The diffusion barriers for chlorine were calculated with the climbing image nudged elastic band (CI-NEB) method^38^. In all CI-NEB calculations, five images are used between the optimized initial and final states. The lattice parameters and all atoms were fixed and only migrating chlorine atom and oxygen atoms on the migration pathways within 3 Å were allowed to relax. A force tolerance of 0.01 eV/Å was used in all CI-NEB calculations.

## Supplementary information

Supplementary Information

Description of Additional Supplementary Files

Video File

Dataset 1

## Data Availability

The data generated in this study are provided in Supplementary Information and Source Data file. The source data of Figs. 1a–c, 2d–f, 3b, e–g, 4b, d, g are provided in the Source Data file.
