# Supplementary material for: An iron-base oxygen-evolution electrode for high-temperature electrolyzers
Source: Nat Commun. 2023 Jan 17;14:253. doi: 10.1038/s41467-023-35904-7 (PMC9845222; doi:10.1038/s41467-023-35904-7)
Supplement: Supplementary file 1 — Supplementary Information [file 41467_2023_35904_MOESM1_ESM.pdf]

Supplementary Information

An iron-base oxygen-evolution electrode for high-temperature electrolyzers

Kaifa Du, Enlai Gao, Chunbo Zhang, Yongsong Ma, Peilin,Wang, Rui Yu, Wenmiao Li, Kaiyuan Zheng, Xinhua Cheng, Diyong Tang, Bowen Deng, Huayi Yin*, Dihua Wang*

*Corresponding author. E-mail: wangdh@whu.edu.cn (D.W.); yinhuayi@whu.edu.cn (H.Y.).

The PDF file includes:

Supplementary Notes 1-3

Supplementary Figures 1-15

Supplementary Tables 1-6

References

Supplementary Notes 1

Basicity of molten salts

The basicity of molten salt is an important index that is defined as:

$$B_{ms} = \lg(a_{O^{2-}}) \qquad (1)$$

where B_{ms} stands for the basicity of molten salt, and $a_{O^{2-}}$ is the activity of O^{2-}. According to the previous research, the activity of O^{2-} could be taken as the sum activity of M_2O in molten carbonates, where M represents alkali metal. The basicity of Li_2CO_3-Na_2CO_3-K_2CO_3 molten salts could be defined as[1]:

$$B_{ms} = \lg(a_{Li_2O} + a_{Na_2O} + a_{Na_2O}) \qquad (2)$$

For Li_2CO_3-Na_2CO_3-K_2CO_3 melt, the carbonates dissociate into M_2O and CO_2,

$$Li_2CO_3 = Li_2O + CO_2 \qquad (3)$$

$$Na_2CO_3 = Na_2O + CO_2 \qquad (4)$$

$$K_2CO_3 = K_2O + CO_2 \qquad (5)$$

where the oxide ion activity should depend on each equilibrium constant and mole fraction of Li_2CO_3, Na_2CO_3, K_2CO_3.

$$\lg(a_{Li_2O}) = -\frac{\Delta G^{\ominus}(3)}{2.3RT} - \lg(P_{CO_2}) + \lg(a_{Li_2CO_3}) \qquad (6)$$

$$\lg(a_{Na_2O}) = -\frac{\Delta G^{\ominus}(4)}{2.3RT} - \lg(P_{CO_2}) + \lg(a_{Na_2CO_3}) \qquad (7)$$

$$\lg(a_{K_2O}) = -\frac{\Delta G^{\ominus}(5)}{2.3RT} - \lg(P_{CO_2}) + \lg(a_{K_2CO_3}) \qquad (8)$$

Where $\Delta G^{\ominus}(3)$, $\Delta G^{\ominus}(4)$, $\Delta G^{\ominus}(5)$ are the Gibbs energy for the reaction (3), reaction (4) and reaction (5); P_{CO_2} is the pressure of CO_2 ; $a_{Li_2CO_3}$, $a_{Na_2CO_3}$ and $a_{K_2CO_3}$ are the activity of Li_2CO_3,

Na_2CO_3 and K_2CO_3. In terms of equation (2), equation (6), equation (7) and equation (8), the basicity of the Li_2CO_3-Na_2CO_3-K_2CO_3 molten salts is equal to[1]:

$$B_{ms}=\lg\left(\exp\left(\frac{\Delta G^{\ominus}(3)}{RT}\right)\times\frac{a_{Li_2CO_3}}{P_{CO_2}}+\exp\left(\frac{\Delta G^{\ominus}(4)}{RT}\right)\times\frac{a_{Na_2CO_3}}{P_{CO_2}}+\exp\left(\frac{\Delta G^{\ominus}(5)}{RT}\right)\times\frac{a_{K_2CO_3}}{P_{CO_2}}\right) \quad (9)$$

In this work, the CO_2 pressure is 0.01 bar; the activity of Li_2CO_3, Na_2CO_3 and K_2CO_3 are 0.435, 0.315 and 0.250. Based on known thermodynamic data (ΔG of reaction (3), reaction (4) and reaction (5)), the basicity of Li_2CO_3-Na_2CO_3-K_2CO_3 melt at various temperature was calculated in this work (Supplementary Fig. 1, Supplementary Table 1).

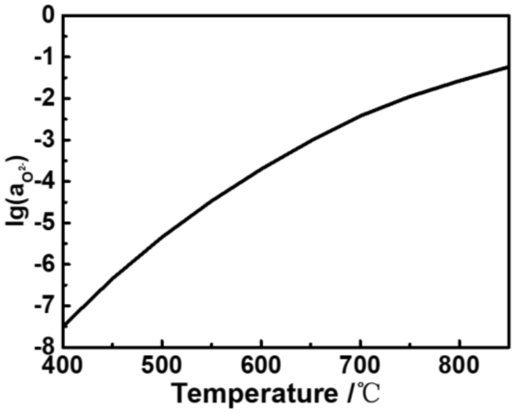

Supplementary Fig. 1. The basicity of Li_2CO_3-Na_2CO_3-K_2CO_3 melt at various temperatures in an argon-1vol.% carbon dioxide mixture gas atmosphere.

Supplementary Table 1. The basicity (B_{ms}) of Li_2CO_3-Na_2CO_3-K_2CO_3 molten salt at various temperatures with an atmosphere of argon-1vol.% CO_2 mixed gas.

Temperature	450 °C	500 °C	550 °C	600 °C	650 °C	700 °C	750 °C
B_{ms}	-6.34	-5.34	-4.47	-3.70	-3.02	-2.42	-1.95

Supplementary Notes 2

DFT calculations

To probe the mechanical compatibility between Fe and $LiFe_5O_8$, we calculated the elastic constants (C_{ij}) of these two materials using the stress-strain method under the framework of density functional theory (DFT). The elastic tensors of Fe and $LiFe_5O_8$ are calculated, respectively, as

$$\begin{bmatrix} 281.9 & 153.0 & 153.0 & 0.0 & 0.0 & 0.0 \\ 153.0 & 281.9 & 153.0 & 0.0 & 0.0 & 0.0 \\ 153.0 & 153.0 & 281.9 & 0.0 & 0.0 & 0.0 \\ 0.0 & 0.0 & 0.0 & 98.4 & 0.0 & 0.0 \\ 0.0 & 0.0 & 0.0 & 0.0 & 98.4 & 0.0 \\ 0.0 & 0.0 & 0.0 & 0.0 & 0.0 & 98.4 \end{bmatrix}$$

and

$$\begin{bmatrix} 271.7 & 173.4 & 173.3 & -0.1 & -0.2 & -0.2 \\ 173.4 & 271.9 & 173.5 & -0.1 & -0.3 & -0.2 \\ 173.3 & 173.5 & 271.9 & -0.1 & -0.2 & -0.2 \\ -0.1 & -0.1 & -0.1 & 66.0 & 0.0 & 0.0 \\ -0.2 & -0.3 & -0.2 & 0.0 & 65.9 & 0.0 \\ -0.2 & -0.2 & -0.2 & 0.0 & 0.0 & 65.9 \end{bmatrix}.$$

These positive definiteness of these elastic tensors[2] indicates that Fe and $LiFe_5O_8$ are mechanically stable. Based on these elastic tensors, the extracted bulk (shear) moduli of Hill average[3] for Fe and $LiFe_5O_8$ are 196.0 (83.1) and 206.2 (58.6) GPa, respectively. The similar moduli indicate the mechanical compatibility of Fe and $LiFe_5O_8$.

Furthermore, the interfacial adhesion between Fe and $LiFe_5O_8$ is of fundamental importance. We calculated the adhesion energy based on the following equation,

$$E_{ad}=(E_{Fe}+E_{LiFe_5O_8}-E_{hyb})/A \quad (10)$$

where E_{hyb}, E_{Fe}, $E_{LiFe_5O_8}$ and A are the energy of $LiFe_5O_8$ adhered on Fe, the energy of Fe slab, the energy of $LiFe_5O_8$ slab, and interfacial area. The calculated adhesion energy E_{ad} is 3.39 J/m^2, indicating

the strong interfacial interaction between Fe and $LiFe_5O_8$. To quantify interfacial bonding characteristics, we calculated the bond orders (BOs) based on a refinement of the density derived electrostatic and chemical approach[4-6]. Supplementary Fig. 2 shows the calculated bond orders at the interface between Fe and $LiFe_5O_8$. These results indicate the strong covalent bonding at the interface. This mechanical robustness of such material interface is the foundation for high-performance applications.

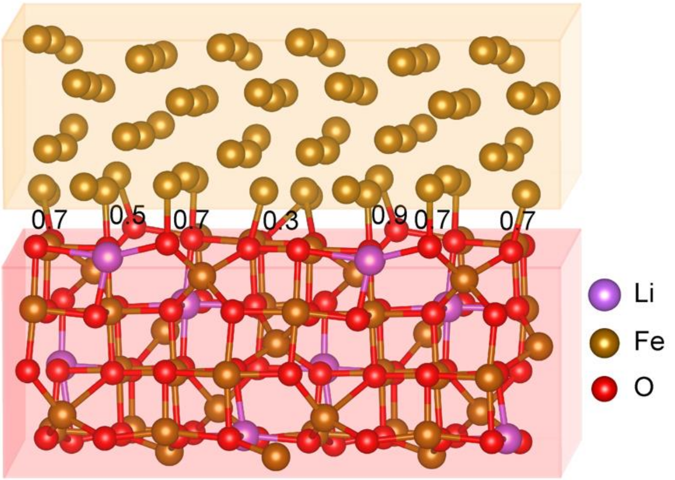

Supplementary Fig. 2. Bond orders at the interface between Fe and $LiFe_5O_8$.

The chemical stability of the iron-base electrode (IBE) can be probed by calculating the diffusion paths of Cl in Fe_2O_3 and $LiFe_5O_8$. The crystal structures of the Fe_2O_3 and $LiFe_5O_8$ are analyzed to find the migration pathways with the possible lowest energy barriers, and the energy profiles of these pathways are calculated by climbing image nudged elastic band (CI-NEB) method[7]. To balance the computational efficiency and accuracy, the oxygen atoms on the migration pathways within 3 Å of Cl atom in Fe_2O_3 and $LiFe_5O_8$ are free to relax. The calculated profiles for Cl diffusing through Fe_2O_3 and $LiFe_5O_8$ are given in Fig. 4a-d**.** The energy barriers for Cl through Fe_2O_3 (1.51 eV) is much lower than that through $LiFe_5O_8$ (2.93 eV), which might be understood by the lower atomic density of Fe_2O_3

(0.09 atom/Å^3) than that of $LiFe_5O_8$ (0.11 atom/Å^3). These results indicate the higher chemical stability of $LiFe_5O_8$ against Cl erosion.

Supplementary Notes 3

Thermodynamic calculation of iron E-lg(a_{Li_2O}) diagram

The electrochemical stability ranges of iron species in electrolyte with different basicity have been evaluated. The standard Gibbs free energy change and equilibrium constant for reactions have been calculated at 650 °C. The thermodynamic data is used to evaluate the anodic oxidation products when the iron is polarized in electrolytes with different basicities. Based on the reactions and corresponding thermodynamic dates (Supplementary Table 2), the E- lg(a_{Li_2O}) diagram of iron at 650 ºC was drawn up.

In the theory developed by Lux and Flood[8-10] the lithium ferrite oxides system could be regarded in terms of the following dissociations:

$$2Fe_5O_8^- = 5Fe_2O_3 + O^{2-} \quad (11)$$

The $LiFe_5O_8$ acts as a base in the supply of oxygen ions, and Fe_2O_3 is their conjugate acid. The lithium oxide serves to “store” oxygen ions, making them available when required for participation in a chemical reaction. The basicity of $LiFe_5O_8$ and Fe_2O_3 can be defined as lg($a_{O^{2-}}$), where $a_{O^{2-}}$ is derived from the dissociation reactions and generally replaced by a_{Li_2O} in the lithium content system:

$$2LiFe_5O_8 = 5Fe_2O_3 + Li_2O \quad (12)$$

$$Fe_2O_3 + LiCl = 2FeCl_3 + 3Li_2O \quad (13)$$

The equilibrium constant of the reaction (12) and (13) are $10^{-10.79}$ and $10^{-7.67}$. When the activity of the

compounds excepting Li_2O is 1, the basicity for the $LiFe_5O_8$ and Fe_2O_3 are -7.67 and -12.91.

Supplementary Table 2. Thermodynamic data of the chemical reaction for the calculation of potential-basicity diagram of iron. The ΔG^{Θ} and K^{Θ} represent respectively the standard Gibbs free energy change and equilibrium constant for reactions at 650 °C.

No.	Reactions	ΔG^{Θ}(kJ/mol)	lg(K^{Θ})
**1**	$FeCl_2 + 2e^- + 2Li^+ = Fe + 2LiCl$	146.34	-8.28
**2**	$FeCl_3 + 3e^- + 3Li^+ = Fe + 3LiCl$	115.126	-6.51
**3**	$FeO + 2Li^+ + 2e^- = Fe + Li_2O$	316.13	-17.89
**4**	$Fe_3O_4 + 8\ Li^+ + 8e^- = 3Fe + 4Li_2O$	1260.63	-17.89
**5**	$Fe_2O_3 + 6Li^+ + 6e^- = 2Fe + 3Li_2O$	914.4	-51.75
**6**	$LiFe_5O_8 + 15\ Li^+ + 15e^- = 5Fe + 8Li_2O$	2353.86	-133.20
**7**	$FeO + 2LiCl = FeCl_2 + Li_2O$	169.79	-9.61
**8**	$Fe_3O_4 + 6LiCl + 2\ Li^+ + 2e^- = 3FeCl_2 + 4Li_2O$	821.61	-46.49
**9**	$FeCl_3 + e^- + Li^+ = FeCl_2 + LiCl$	-31.21	1.77
**10**	$Fe_2O_3 + 4LiCl + 2\ Li^+ + 2e- = 2FeCl_2 + 3Li_2O$	621.76	-35.18
**11**	$LiFe_5O_8 + 10LiCl + 5\ Li^+ + 5e^- = 5FeCl_2 + 8Li_2O$	1622.17	-91.80
**12**	$LiFeO_2 + 2LiCl + Li^+ + e^- = FeCl_2 + 2Li_2O$	362.58	-20.52
**13**	$Fe_3O_4 + 2\ Li^+ + 2e^- = 3FeO + Li_2O$	312.25	-17.67
**14**	$FeCl_3 + Li_2O + Li^+ + e^- = FeO + 3LiCl$	-201.00	11.37
**15**	$Fe_2O_3 + 2Li^+ + 2e^- = 2FeO + Li_2O$	282.19	-15.97
**16**	$LiFe_5O_8 + 5Li^+ + 5e^- = 5FeO + 3Li_2O$	773.23	-43.75
**17**	$3FeCl_3 + 4Li_2O + Li^+ + e^- = Fe_3O_4 + 9LiCl$	-915.25	51.79
**18**	$3Fe_2O_3 + 2\ Li^+ + 2e^- = 2Fe_3O_4 + Li_2O$	222.07	-12.57
**19**	$3LiFe_5O_8 + 5\ Li^+ + 5e^- = 5Fe_3O_4 + 4Li_2O$	758.446	-42.92
**20**	$Fe_2O_3 + 6LiCl = 2FeCl_3 + 3Li_2O$	684.19	-38.72
**21**	$LiFe_5O_8 + 15LiCl = 5FeCl_3 + 8Li_2O$	1778.23	-100.63
**22**	$2LiFe_5O_8 = 5Fe_2O_3 + Li_2O$	135.52	-7.67

**23**	$O_2(g) + 4Li^+ + 4e^- = 2Li_2O$	224.08	-12.68

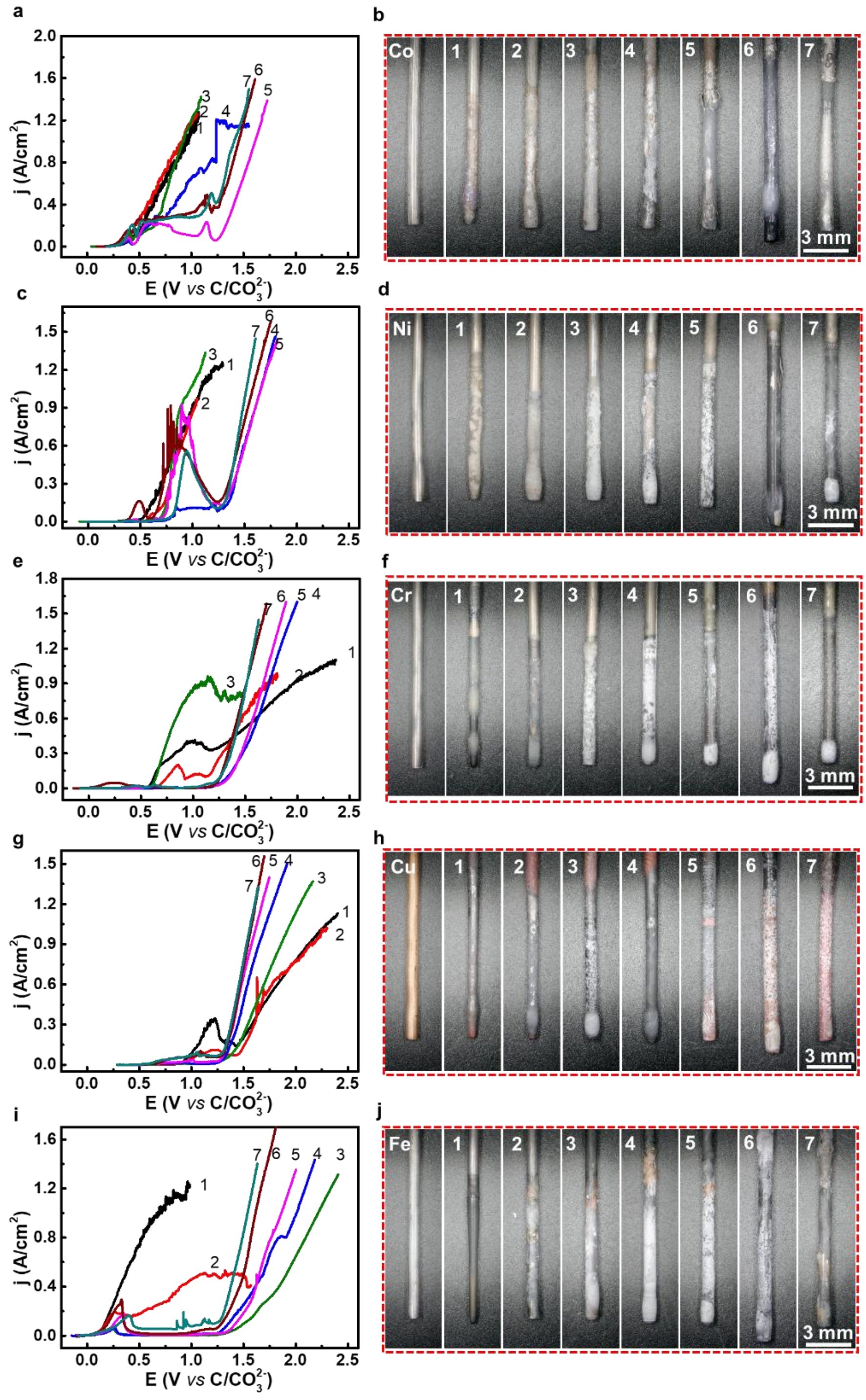

Supplementary Fig. 3. Anodic polarization behaviors of metal electrodes in molten Li_2CO_3-Na_2CO_3-K_2CO_3 with various B_{ms}. (**a, c, e, g, i**) Anodic polarization curves of cobalt (**a**), nickel (**c**),

chromium (**e**), copper (**g**) and iron (**i**) in molten Li_2CO_3-Na_2CO_3-K_2CO_3 with various B_{ms} (1, B_{ms} = -6.34 (450°C); 2, B_{ms} = -5.34 (500°C); 3, B_{ms} = -4.47 (550°C); 4, B_{ms} = -3.70 (600°C); 5, B_{ms} = -3.02 (650°C); 6, B_{ms} = -2.42 (700°C); 7, B_{ms} = -1.95 (750°C)). (**b, d, f, h, g)** Optical graphs of cobalt (**b**), nickel (**d**), chromium (**f**), copper (**h**) and iron (**g**) before and after anodic polarization. The metal electrodes of cobalt, nickel, chromium, copper, iron show active dissolution behavior at low B_{ms}, but passivation behavior at high B_{ms}. The dissolution-passivation transition B_{ms} are -3.02 (cobalt), -3.70 (nickel, chromium and copper), -4.47 (iron).

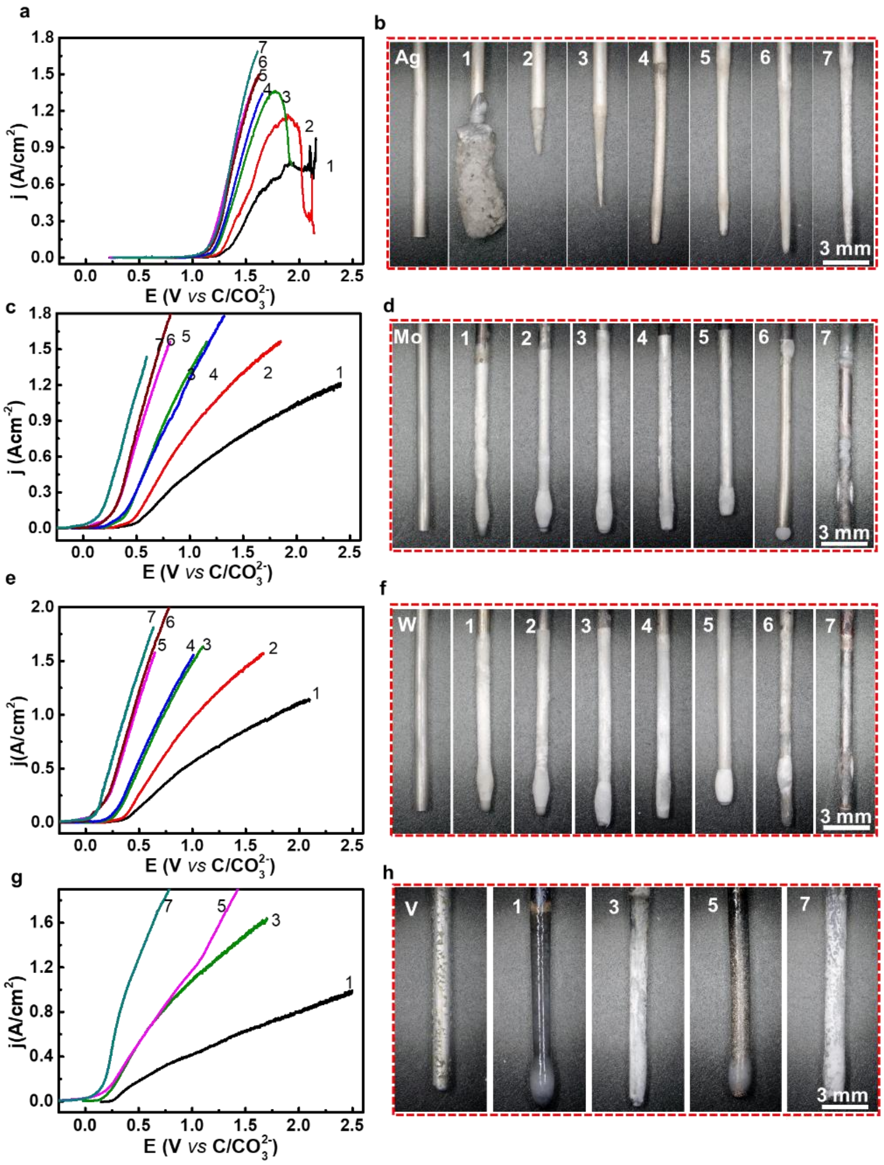

Supplementary Fig. 4. Anodic polarization behaviors of metal electrodes in molten Li_2CO_3-Na_2CO_3-K_2CO_3 with various B_{ms}. (**a, c, e, g**) Anodic polarization curves of silver (**a**), molybdenum (**c**), tungsten (**e**) and vanadium (**g**) in molten Li_2CO_3-Na_2CO_3-K_2CO_3 with various B_{ms} (1, B_{ms} = -6.34 (450°C); 2, B_{ms} = -5.34 (500°C); 3, B_{ms} = -4.47 (550°C); 4, B_{ms} = -3.70 (600°C); 5, B_{ms} = -3.02 (650°C); 6, B_{ms} = -2.42 (700°C); 7, B_{ms} = -1.95 (750°C)). (**b, d, f, h**) Optical graphs of silver (**b**), molybdenum (**d**), tungsten (**f**) and vanadium (**h**) before and after anodic polarization. The metal electrodes of silver, molybdenum, tungsten and vanadium show active dissolution behavior with a B_{ms} range of -6.34 – -1.95.

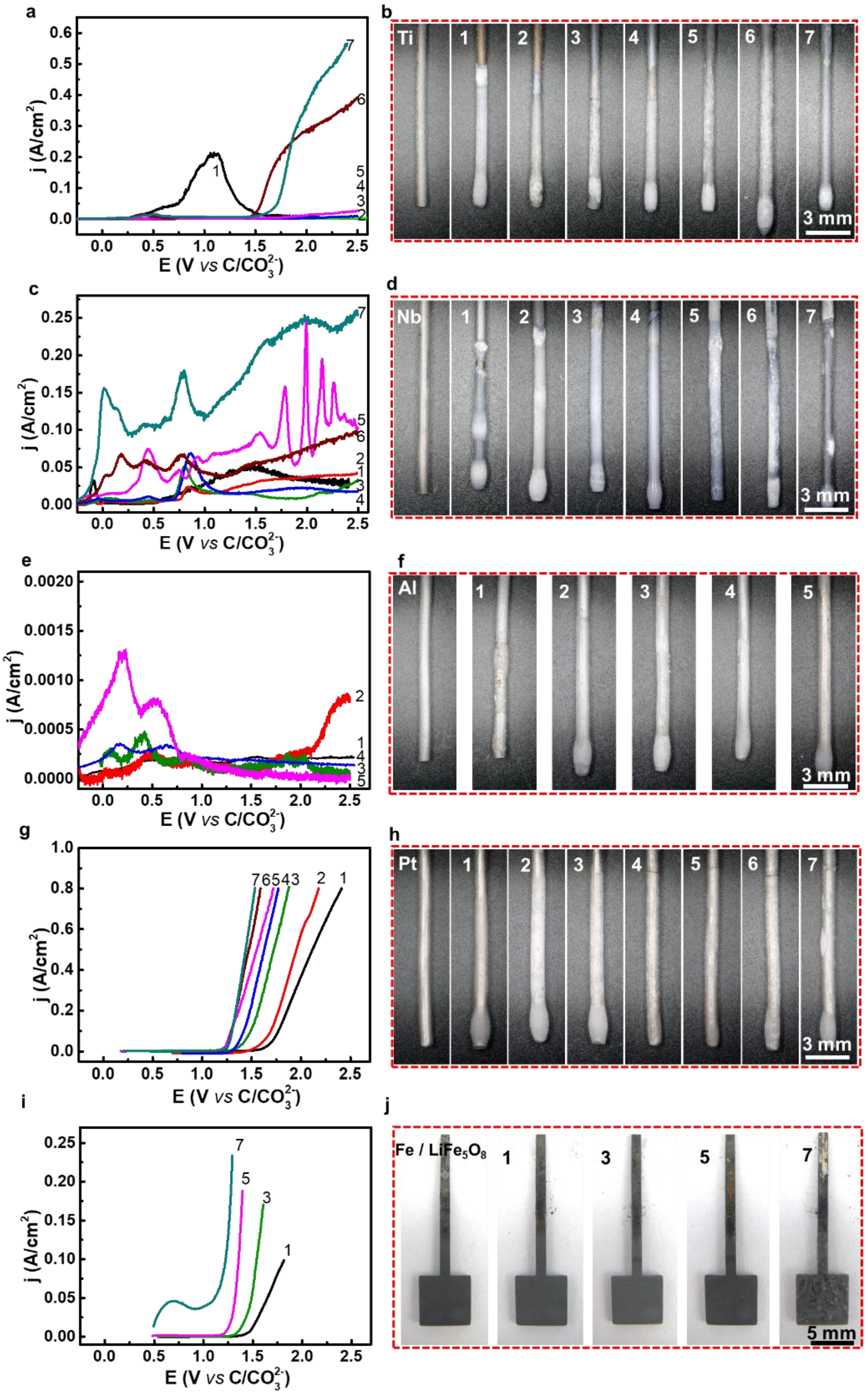

Supplementary Fig. 5. Anodic polarization behaviors of metal electrodes in molten Li_2CO_3-Na_2CO_3-K_2CO_3 with various B_{ms}. (**a, c, e, g, i**) Anodic polarization curves of titanium (**a**), niobium (**c**), aluminum (**E**), platinum (**g**) and lithium ferrite coated iron (**i**) in molten Li_2CO_3-Na_2CO_3-K_2CO_3 with various B_{ms} (1, B_{ms} = -6.34 (450°C); 2, B_{ms} = -5.34 (500°C); 3, B_{ms} = -4.47 (550°C); 4, B_{ms} = -

3.70 (600°C); 5, B_{ms} = -3.02 (650°C); 6, B_{ms} = -2.42 (700°C); 7, B_{ms} = -1.95 (750°C)). (**b, d, f, h, j**) Optical graphs of titanium (**b**), niobium (**d**), aluminum (**f**), platinum(**h**) and lithium ferrite coated iron (**j**) before and after anodic polarization. The metal electrodes of titanium and niobium show passivation behavior with low B_{ms}, but active dissolution behavior with high B_{ms}. The corresponding passivation-dissolution transition B_{ms} is -2.42 (titanium), and -3.02(niobium). The metal electrodes of aluminum show passivation behavior with a B_{ms} range of -6.34 – -3.02. The metal electrodes of platinum and lithium ferrite coated iron show passivation behavior with a B_{ms} range of -6.34–-1.95 and both of them have a good oxygen evolution performance.

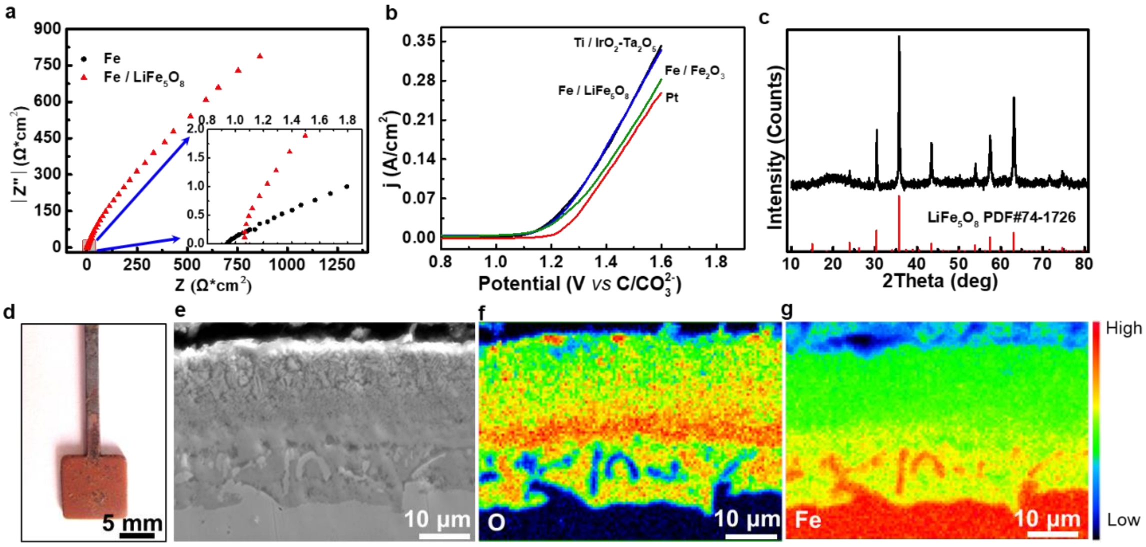

Supplementary Fig. 6. High temperature oxygen evolution (HT-OER) performance and stability of iron electrode with LFO film. (**a**) Electrochemical impedance spectroscopy of the iron anodes (with and without LFO film) in molten Li_2CO_3-Na_2CO_3-K_2CO_3 at 450 °C. The circuit ohmic resistance of the iron anodes with and without LFO film are 1.07 Ω·cm^2 and 0.95 Ω·cm^2. (**b**) Anodic polarization curves of commercial Ti/ IrO_2-Ta_2O_5, iron electrode with LFO film, iron with Fe_2O_3 film and platinum electrodes. The iron electrodes were anodically oxidized for 10 min with a current density of 100 mA/cm^2 in molten Li_2CO_3-Na_2CO_3-K_2CO_3 at 650 °C to form LFO film, and oxidized for 2 h in the air at 650 °C to form Fe_2O_3 film. The Ti/ IrO_2-Ta_2O_5 anode (IR-MMO) was manufactured by Magneto Special Anodes Co. Ltd., which generally exhibits an excellent catalytic performance of OER. The HT-OER catalytic performance of the iron electrode with LFO film is similar to the commercial Ti/IrO_2-Ta_2O_5 electrode, and better than the iron electrode with Fe_2O_3 film and platinum electrode. (**c-g**) Characterization of the iron electrode after electrolysis for in molten carbonates at 450 °C. (**c**) XRD pattern, (**d**) Optical graphs, (**e**) SEM micrograph, and (**e, f, g**) EDS mapping of Fe and O of cross-section, of the iron anode with LFO film after constant current electrolysis at a current density of 100 mA/cm^2 in molten Li_2CO_3-Na_2CO_3-K_2CO_3 at 450 °C for 100 h.

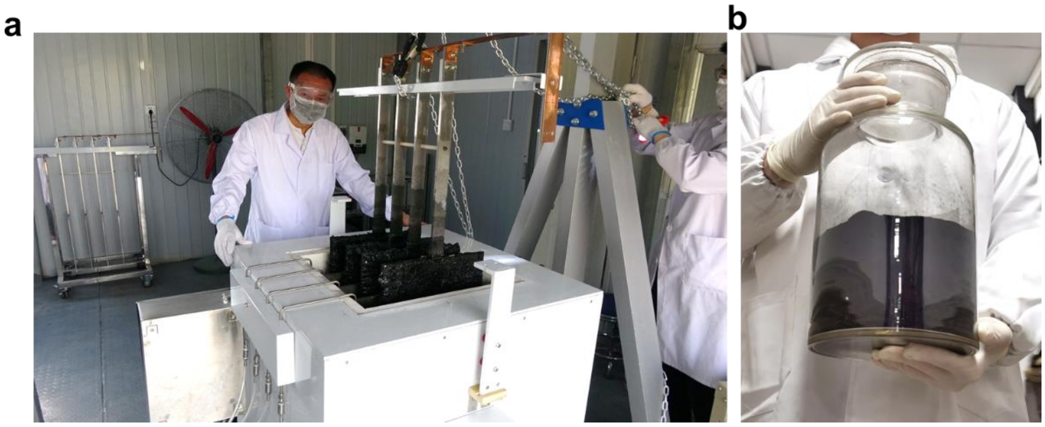

Supplementary Fig. 7. Kiloampere-grade scale-up of the molten salt CO_2 capture and electrochemical transformation process with the use of iron electrode. (**a**) Optical graph of the scale-up device. There were three 350 mm×400 mm×5 mm iron electrode with LFO film as the anodes and four 350 mm×400 mm×5 mm nickel cathodes, which were arranged alternately in the electrolytic tank. 187 kg of anhydrous Li_2CO_3-Na_2CO_3-K_2CO_3 (mixed in a molar ratio of 43.5: 31.5: 25.0) were melted as the electrolyte at 650 ºC. This device was conducted with a current of 1160 A, which can adsorb 5.0 Nm^3 of CO_2 and produce 2.7 kg of carbon materials and 5.0 Nm^3 of O_2 per day. (**b**) Optical graph of the carbon products transformed from CO_2.

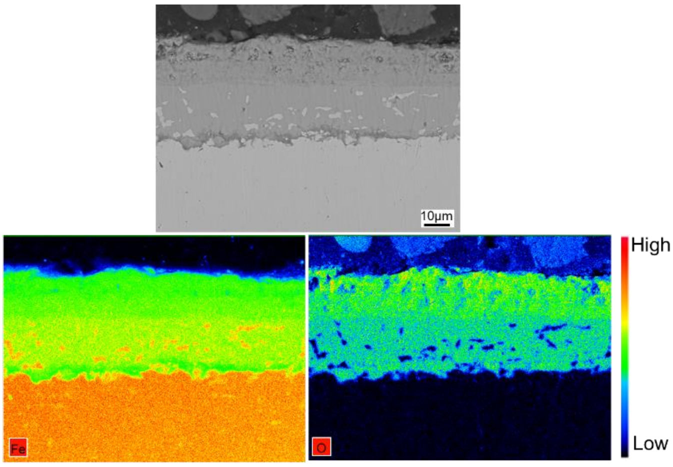

Supplementary Fig. 8. SEM image and EPMA mapping of the cross-section of the Fe electrode after anodic polarization for 2 h at 100 mA cm^{-2} in Li_2CO_3-Na_2CO_3-K_2CO_3 at 450 °C. The thickness of oxide film is 37 μm.

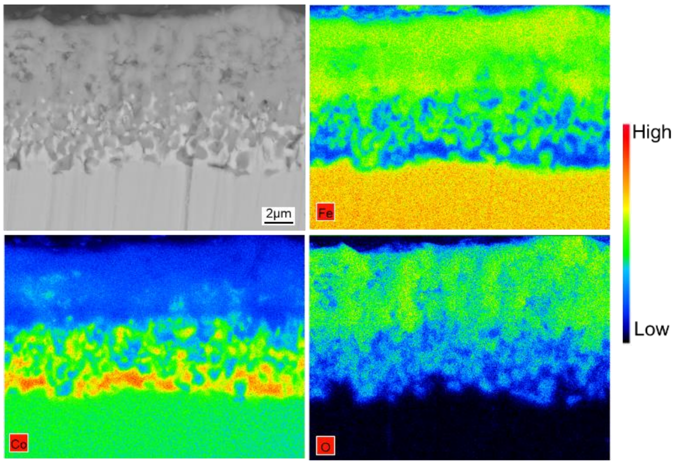

Supplementary Fig. 9.SEM image and EPMA mapping of the cross-section of the Fe-36Co electrode after anodic polarization for 2 h at 100 mA cm^{-2} in Li_2CO_3-Na_2CO_3-K_2CO_3 at 650 °C. The thickness of oxide film is 6 μm.

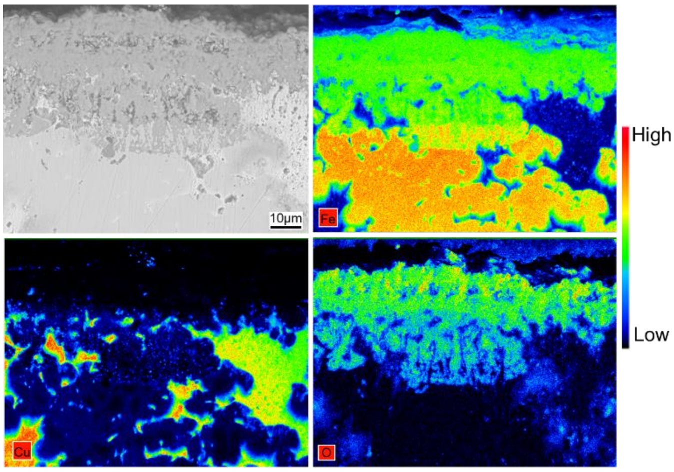

Supplementary Fig. 10. SEM image and EPMA mapping of the cross-section of the Fe-36Cu electrode after anodic polarization for 2 h at 100 mA cm^{-2} in Li_2CO_3-Na_2CO_3-K_2CO_3 at 650 ºC. The thickness of oxide film is 56 μm.

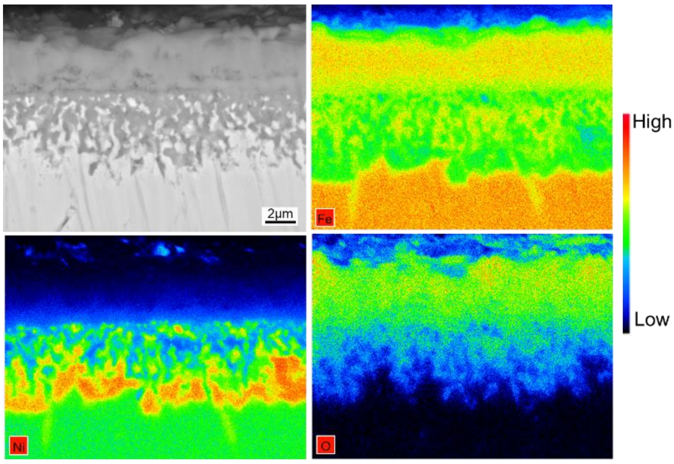

Supplementary Fig. 11. SEM image and EPMA mapping of the cross-section of the Fe-36Ni electrode after anodic polarization for 2 h at 100 mA cm^{-2} in Li_2CO_3-Na_2CO_3-K_2CO_3 at 650 ºC. The thickness of oxide film is 4 μm. The least thickness of $LiFe_5O_8$ film on Fe-36Ni indicates the best high-temperature oxidation resistance of Fe-36Ni.

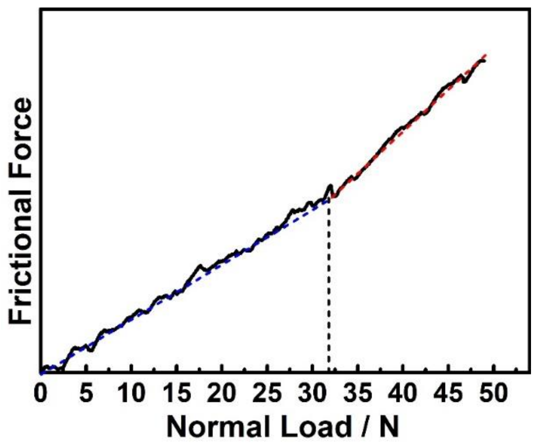

Supplementary Fig. 12. Binding force test of the oxide film of pre-oxidized Fe-36Ni electrode. The normal load of the break of oxide film could reach 32 N , indicating that the oxide film has a good binding force.

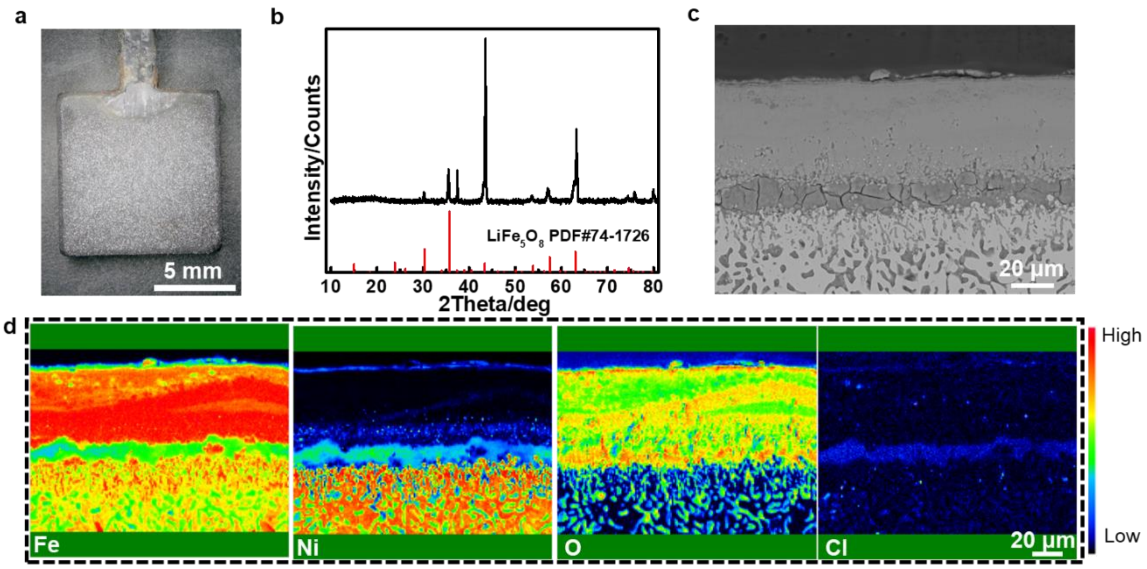

Supplementary Fig. 13. Characterization of the Fe-36Ni electrode after electrolysis in molten lithium chloride at 650 °C. (**a**) Optical graphs, (**b**) XRD spectra, (**c**) SEM micrograph (SE), (**d**) EPMA mapping (Fe, Ni, O, Cl) of the cross-section of Fe-36Ni anode with LFO film after constant current electrolysis in molten LiCl-1.5wt.% Li_2O at 650 °C for 20 days (100 mA/cm^2). The dimension of the anode and the composition of the oxide film maintains its original shape and chemical composition.

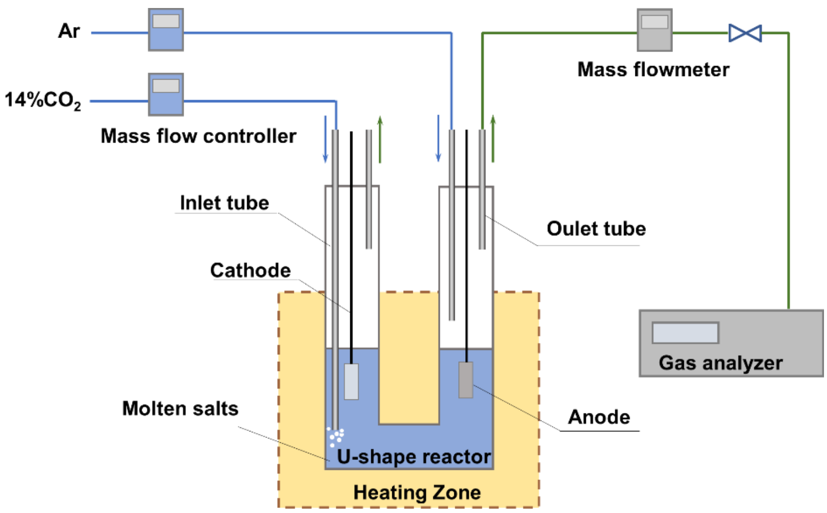

Supplementary Fig. 14. The schematic of U-shape reactor equipped with an in-situ gas analyzer.

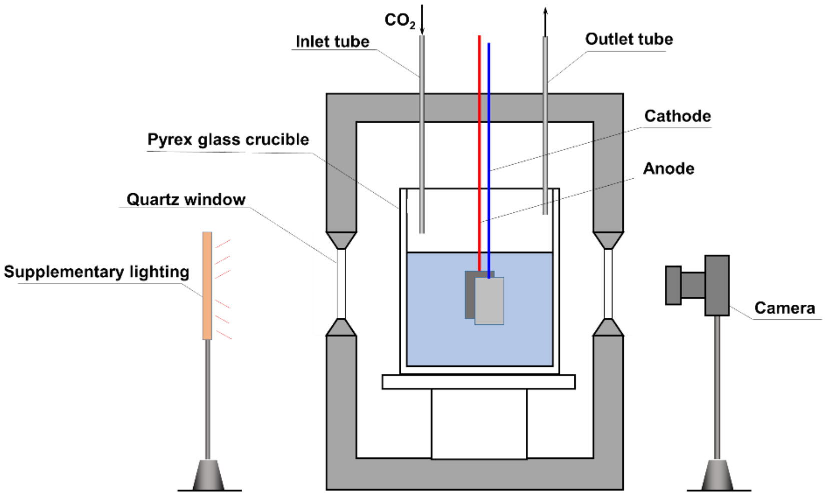

Supplementary Fig. 15. The schematic of the transparent electrolysis cell.

Supplementary Table 3. The results of anodic polarization tests of metals in Li_2CO_3-Na_2CO_3-K_2CO_3 molten salt at various basicities. "D" and "P" are representing active dissolution and passivation behaviors.

**Metals\B_{ms}**	**-6.34**	**-5.34**	**-4.47**	**-3.70**	**-3.02**	**-2.42**	**-1.95**
Ag	D	D	D	D	D	D	D
Cr	D	D	D	P	P	P	P
Co	D	D	D	D	P	P	P
Cu	D	D	D	P	P	P	P
Ni	D	D	D	P	P	P	P
Fe	D	D	P	P	P	P	P
Pre-oxidation Fe	P	/	P	/	P	/	P
Al	P	P	P	P	P	/	/
Pt	P	P	P	P	P	P	P
Ti	P	P	P	P	P	D	D
Nb	P	P	P	P	D	D	D
V	D	/	D	/	D	/	D
W	D	D	D	D	D	D	D
Mo	D	D	D	D	D	D	D

Supplementary Table 4. Charge, ionic radius (nm) and ionic potential of the metal ions[11].

Ion	Charge	Ionic radius（nm）		Ionic potential (nm^{-1})	
		**High spin**	**Low spin**	**High spin**	**Low spin**
Ag	1	0.1150		8.6957	
Cu	1	0.077		12.9870	
Cr	2	0.0800	0.0730	25.000	27.3973
Co	2	0.0745	0.0650	26.8456	30.7692
Ni	2	0.0690		28.9855	
Fe	2	0.0645	0.0550	31.0078	36.3636
V	2	0.0790		25.3165	
Ti	2	0.0860		23.2558	
Pt	2	0.0800		25.0000	
Ag	2	0.0940		21.2766	
Cu	2	0.0730		27.3973	
Ti	3	0.0670		44.7761	
Fe	3	0.0645	0.0550	46.5116	54.5455
V	3	0.0640		46.8750	
Cr	3	0.0615		48.7805	
Co	3	0.0610	0.0545	49.1803	55.0459
Ni	3	0.0600	0.0560	50.0000	53.8714
Al	3	0.0535		56.0748	
Nb	4	0.0680		58.8235	
Mo	4	0.0650		61.5385	
Ir	4	0.0625		64.0000	
Pt	4	0.0625		64.0000	

Ti	4	0.0605	66.1157
V	4	0.0580	68.9655
W	4	0.066	60.6061
Nb	5	0.0640	78.1250
Ta	5	0.0640	78.1250
V	5	0.0540	92.5926
Cr	6	0.044	136.3636
W	6	0.0600	100.0000
Mo	6	0.0590	101.6949

Supplementary Table 5. Standard equilibrium constant of the M^{n+} and MO_x^{y-} dissolution reactions of metal oxides at 650 °C. K^{Θ} is the standard equilibrium, which was calculated by HSC Chemistry 6.0; K' is the normalized standard equilibrium ($\lg(K') = \frac{\lg(K^{\Theta})}{n(O^{2-})}$); K is the normalized equilibrium in the molten salts with a O^{2-} activity of $10^{-3.02}$ ($K = \frac{K'}{a\,(O^{2-})}$); where the $n(O^{2-})$ is the number of the Li_2O in the reaction equation and the $a(O^{2-})$ is O^{2-} activity of the molten carbonates at 650 °C.

**Oxide**	**Reaction**	**n(O^{2-})**	**lg(K$^{\Theta}$)**	**lg(K')**	**lg(K)**
VO	$VO + 2Li^+ = V^{2+} + Li_2O$	1	-23.01	-23.01	-26.03
V_2O_3	$V_2O_3 + 6Li^+ = 2V^{3+} + 3Li_2O$	3	-76.66	-25.55	-28.57
Cr_2O_3	$Cr_2O_3 + 6Li^+ = 2Cr^{3+} + 3Li_2O$	3	-86.81	-28.94	-31.96
FeO	$FeO + 2Li^+ = Fe^{2+} + Li_2O$	1	-20.40	-20.40	-23.42
Fe_2O_3	$Fe_2O_3 + 6Li^+ = 2Fe^{3+} + 3Li_2O$	3	-85.58	-28.53	-31.55
CoO	$CoO + 2Li^+ = Co^{2+} + Li_2O$	1	-21.04	-21.04	-24.06
Co_3O_4	$Co_3O_4 + 8Li^+ = 2Co^{3+} + Co^{2+} + 4Li_2O$	4	-116.82	-29.21	-32.23
NiO	$NiO + 2Li^+ = Ni^{2+} + Li_2O$	1	-21.76	-21.76	-24.78
Cu_2O	$Cu_2O + 2Li^+ = 2Cu^+ + Li_2O$	1	-14.65	-14.65	-17.67
CuO	$CuO + 2Li^+ = Cu^{2+} + Li_2O$	1	-21.38	-21.38	-24.40
Al_2O_3	$Al_2O_3 + 6Li^+ = 2Al^{3+} + 3Li_2O$	3	-86.01	-28.67	-31.69
Ag_2O	$Ag_2O + 2Li^+ = 2Ag^+ + Li_2O$	1	-7.46	-7.46	-10.48
AgO	$AgO + 2Li^+ = Ag^{2+} + Li_2O$	1	-24.46	-24.46	-27.48
V_2O_5	$V_2O_5 + Li_2O = 2VO_3^- + 2Li^+$	1	-57.89	-19.30	-22.32
Cr_2O_3	$Cr_2O_3 + Li_2O = 2CrO_2^- + 2Li^+$	1	-30.74	-30.74	-33.76
CrO_3	$CrO_3 + Li_2O = CrO_4^{2-} + 2Li^+$	1	-13.03	-13.03	-16.05
Fe_2O_3	$Fe_2O_3 + Li_2O = 2FeO_2^- + 2Li^+$	1	-25.15	-25.15	-28.17

CoO	$CoO + Li_2O = CoO_2^{2-} + 2Li^+$	1	-29.36	-29.36	-32.38
NiO	$NiO + Li_2O = NiO_2^{2-} + 2Li^+$	1	-28.95	-28.95	-31.97
CuO	$CuO + Li_2O = CuO^{2-} + 2Li^+$	1	-28.24	-28.24	-31.26
Al_2O_3	$Al_2O_3 + Li_2O = 2AlO_2^- + 2Li^+$	1	-23.57	-23.57	-26.59
Nb_2O_5	$Nb_2O_5 + Li_2O = 2NbO_3^- + 2Li^+$	1	-23.44	-23.44	-26.46
WO_3	$WO_3 + Li_2O = WO_4^{2-} + 2Li^+$	1	-16.10	-16.10	-19.12
MoO_3	$MoO_3 + Li_2O = MoO_4^{2-} + 2Li^+$	1	-16.18	-16.18	-19.20

Supplementary Table 6. Potentials of the formation of metal oxides in molten salt (650 °C). E^{Θ} is the standard potential, which was calculated by HSC Chemistry 6.0; and E is the potential in molten salts with a O^{2-} activity of $10^{-3.02}$ ($E = E^{\Theta} + \frac{n(O^{2-})}{n(e^{-})F}\ln(a(O^{2-}))$); where $n(O^{2-})$ and $n(e^{-})$ are the number of Li_2O and the number of charge in the reaction equation respectively, and $a(O^{2-})$ is O^{2-} activity of the molten carbonates at 650 °C, and F is Faraday constant.

**Metal**	**Oxide**	**Reaction**	**E^{Θ} (V *vs* C/CO_3^{2-})**	**E(V *vs* C/CO_3^{2-})**
**OER reaction**		$2Li_2O - 4e^- = O_2(g) + 4Li^+$	1.238	1.100
**Reference electrode reaction**		$Li_2CO_3 + Li^+ + 4e^- = 3Li_2O + C$	0	
Cu	Cu_2O	$2Cu + Li_2O - 2e^- = Cu_2O + 2Li^+$	0.716	0.578
	CuO	$Cu_2O + Li_2O - 2e^- = 2CuO + 2Li^+$	1.010	0.872
Ni	NiO	$Ni + Li_2O - 2e^- = NiO + 2Li^+$	0.435	0.297
Co	CoO	$Co + Li_2O - 2e^- = CoO + 2Li^+$	0.365	0.227
	Co_3O_4	$3CoO + Li_2O - 2e^- = Co_3O_4 + 2Li^+$	0.991	0.853
Fe	FeO	$Fe + Li_2O - 2e^- = FeO + 2Li^+$	0.182	0.044
	Fe_3O_4	$FeO + Li_2O - 2e^- = Fe_3O_4 + 2Li^+$	0.202	0.064
	Fe_2O_3	$2Fe_3O_4 + Li_2O - 2e^- = 3Fe_2O_3 + 2Li^+$	0.670	0.532
Cr	Cr_2O_3	$2Cr + 3\ Li_2O - 6e^- = Cr_2O_3 + 6Li^+$	-0.299	-0.437
	CrO_3	$Cr_2O_3 + 3\ Li_2O - 6e^- = 2CrO_3 + 6Li^+$	1.535	1.397
V	VO	$V + Li_2O - 2e^- = VO + 2Li^+$	-0.564	-0.702
	V_2O_3	$2V + 3\ Li_2O - 6e^- = V_2O_3 + 6Li^+$	-0.447	-0.585
	VO_2	$V_2O_3 + Li_2O - 2e^- = 2VO_2 + 2Li^+$	0.536	0.398
	V_2O_5	$2VO_2 + Li_2O - 2e^- = V_2O_5 + 2Li^+$	1.052	0.914
Ti	TiO	$Ti + Li_2O - 2e^- = TiO + 2Li^+$	-1.111	-1.249
	Ti_2O_3	$2TiO + Li_2O - 2e^- = Ti_2O_3 + 2Li^+$	-0.598	-0.736

	TiO_2	$Ti_2O_3 + Li_2O - 2e- = 2TiO_2 + 2Li^+$	-0.27	-0.408
Al	Al_2O_3	$2Al + 3\ Li_2O - 6e- = Al_2O_3 + 6Li^+$	-1.156	-1.294
Nb	NbO	$Nb + Li_2O - 2e- = NbO + 2Li^+$	-0.50	-0.638
	NbO_2	$NbO + Li_2O - 2e- = NbO_2 + 2Li^+$	-0.268	-0.406
	Nb_2O_5	$2NbO_2 + Li_2O - 2e- = Nb_2O_5 + 2Li^+$	-0.015	-0.153
W	WO_2	$W + 2\ Li_2O - 4e- = WO_2 + 4Li^+$	0.149	0.011
	WO_3	$WO_2 + Li_2O - 2e- = WO_3 + 2Li^+$	0.288	0.15
Mo	MoO_2	$Mo + 2\ Li_2O - 4e- = MoO_2 + 4Li^+$	0.153	0.015
	MoO_3	$MoO_2 + Li_2O - 2e- = MoO_3 + 2Li^+$	0.762	0.624
Pt	PtO	$Pt + Li_2O - 2e- = PtO + 2Li^+$	1.274	1.136
	PtO_2	$PtO + Li_2O - 2e- = PtO_2 + 2Li^+$	1.317	1.179
Ag	Ag_2O	$2Ag + Li_2O - 2e- = Ag_2O+ 2Li^+$	1.384	1.246
	AgO	$Ag + Li_2O - 2e- = AgO + 2Li^+$	1.799	1.661

References

1. Lee, S. & Staehle, R. W. Test of a dual electrode galvanic cell in binary carbonate melt. *Mater. Corros.* **49**, 7–12 (2002).
2. Born, M., Huang, K. & Lax, M. Dynamical theory of crystal lattices. *Am. J. Phys.* **23**, 474–474 (1955).
3. Hill, R. The elastic behaviour of a crystalline aggregate. *Proc. Phys. Soc. Sect. A* **65**, 349–354 (1952).
4. Manz, T. A. & Limas, N. G. Introducing DDEC6 atomic population analysis: Part 1. Charge partitioning theory and methodology. *RSC Adv.* **6**, 47771–47801 (2016).
5. Limas, N. G. & Manz, T. A. Introducing DDEC6 atomic population analysis: Part 2. Computed results for a wide range of periodic and nonperiodic materials. *RSC Adv.* **6**, 45727–45747 (2016).

6. Manz, T. A. Introducing DDEC6 atomic population analysis: Part 3. Comprehensive method to compute bond orders. *RSC Adv.* **7**, 45552–45581 (2017).

7. Henkelman, G., Uberuaga, B. P. & Jónsson, H. Climbing image nudged elastic band method for finding saddle points and minimum energy paths. *J. Chem. Phys.* **113**, 9901–9904 (2000).

8. H, L. "Säuren" und "Basen" im schmelzfluss: Die bestimmung der sauerstoffionen-konzentration. *Zeitschrift für Elektrochemie und Angew. Phys. Chemie* **725**, 303-309. (1937).

9. Flood H, F. T. The acidic and basic properties of oxides. *Acta Chem. Scand* **1**, 592–604 (1947).

10. Duffy, J. A. Optical Basicity: A practical acid-base theory for oxides and oxyanions. *J. Chem. Educ.* **73**, 1138 (2009).

11. Zhao, C. *et al.* Rational design of layered oxide materials for sodium-ion batteries. *Science.* **370**, 708–712 (2020).
