# Supplementary material for: An iron-base oxygen-evolution electrode for high-temperature electrolyzers
Source: Nat Commun. 2023 Jan 17;14:253. doi: 10.1038/s41467-023-35904-7 (PMC9845222; doi:10.1038/s41467-023-35904-7)
Supplement: Supplementary file 2 — Description of Additional Supplementary Files [file 41467_2023_35904_MOESM2_ESM.pdf]

Legends for Video:

Supplementary Video. Oxygen gas evolution on a pre-oxidized iron anode during electrolysis at a current density of 100mA cm^{-2} in molten Li_2CO_3-Na_2CO_3-K_2CO_3 at 450 ^oC using a transparent cell with a quartz window.

Legends for Dataset 1:

Source Data. Source data of Fig. 1a-c, 2d-f, 3b, 3e-g, 4b, 4d, 4g.
